# Frictional Wear and Corrosion Behavior of AlCoCrFeNi High-Entropy Alloy Coatings Synthesized by Atmospheric Plasma Spraying

**DOI:** 10.3390/e22070740

**Published:** 2020-07-04

**Authors:** Yongkun Mu, Liangbo Zhang, Long Xu, Kondagokuldoss Prashanth, Nizhen Zhang, Xindi Ma, Yuefei Jia, Yulai Xu, Yandong Jia, Gang Wang

**Affiliations:** 1 Institute of Materials, Shanghai University, Shanghai 200444, China; muyongkun@shu.edu.cn (Y.M.); Liangbo0703@shu.edu.cn (L.Z.); L.Xu@shu.edu.cn (L.X.); prashanth.konda@taltech.ee (K.P.); dadani@shu.edu.cn (N.Z.); xindima@shu.edu.cn (X.M.); YuefeiJia@shu.edu.cn (Y.J.); yulaixu@shu.edu.cn (Y.X.); 2 Department of Mechanical and Industrial Engineering, Tallinn University of Technology, 19086 Tallinn, Estonia; 3 Erich Schmid Institute of Materials Science, Austrian Academy of Sciences, Jahnstraße 12, A-8700 Leoben, Austria; 4 Centre for Biomaterials, Cellular and Molecular Theranostics, School of Mechanical Engineering, Vellore Institute of Technology, Vellore 632014, Tamil Nadu, India

**Keywords:** high-entropy alloy, coatings, atmospheric plasma spraying, frictional wear, corrosion

## Abstract

High-entropy alloy coatings (HEAC) exhibit good frictional wear and corrosion resistances, which are of importance for structure materials. In this study, the microstructure, surface morphology, hardness, frictional wear and corrosion resistance of an AlCoCrFeNi high-entropy alloy coating synthesized by atmospheric plasma spraying (APS) were investigated. The frictional wear and corrosion resistance of the coating are simultaneously improved with an increase of the power of APS. The influence of the APS process on the microstructure and mechanical behavior is elucidated. The mechanisms of frictional wear and corrosion behavior of the AlCoCrFeNi HEAC are discussed in detail.

## 1. Introduction

High-entropy alloys (HEAs), comprised of at least five principal elements with the atomic percentage of each element varying from 5 to 35%, have attracted increasing attention because of their appealing properties and potential applications [1,2,3]. The AlCoCrFeNi-M (M = Cu, Mn, Ti, B or Si, etc.) alloy system is one of the most notable HEAs to date, which has attracted the interest of many researchers since it exhibits attractive properties, such as high strength, high fatigue resistance, high fracture toughness, high-temperature oxidation resistance, high corrosion resistance, unique electrical and magnetic properties, etc. [4,5,6,7,8,9,10,11]. Thus, AlCoCrFeNi-M HEAs are potential candidates for structural and functional materials [12,13,14,15]. Apart from bulk HEAs consolidated from liquids, HEAs can also be deposited as a surface coating with a high performance. As such, investigations of HEA coatings (HEACs) is one of the upcoming research fields where there is a growing interest among the material scientists and engineers. To date, numerous technologies have been applied to fabricate HEACs [16,17], such as magnetron sputtering [18,19], laser cladding [20,21], spraying [22,23,24], electrodeposition [25] and plasma cladding [26], which have proved that HEACs can exhibit excellent mechanical and physical properties, for instance, high hardness, wear and corrosion resistance and good and appealing electrical and magnetic properties, as well as excellent performance at high temperatures [16,17].

Both abrasion and corrosion resistance are important properties of coatings. Recently, the frictional wear and corrosion behavior of AlCoCrFeNi-M HEACs have been reported [20,27,28,29]; AlCoCrFeCu-X_0.5_ HEACs were fabricated by laser [20], in which an AlCoCrFeCuSi_0.5_ HEAC exhibited the lowest corrosion rate. A CoCrFeNiMn HEAC manufactured by a laser surface alloying technique contains columnar dendrites that are of a face center cubic (FCC) phase, which exhibits a good corrosion resistance in a 3.5 wt.% NaCl and 0.5 M H_2_SO_4_ solution [27]. On the other hand, the wear resistance of HEACs have also been investigated [28,29]. In an Al_0.5_CoCrFeNiSi HEAC synthesized by atmospheric plasma spraying or laser re-melting, Si is of importance affecting the wear resistance of the coating [28]. Furthermore, when an Mg-based alloy (AZ91D) is coated by an Al_0.5_CoCrFeCuNi HEAC through laser cladding, the wear rate of the Mg-based alloy is improved approximately 2.5 times, and the corrosion resistance is also significantly improved [29].

However, the preparation of HEACs by thermal spraying has received less attention. Atmospheric plasma spraying (APS), as a well-known thermal spraying technique due to the simplicity in selecting the kinds of powder and high production efficiency, is useful for fabricating HEACs. Therefore, in the current study, an AlCoCrFeNi HEAC is manufactured by APS. The frictional wear and corrosion resistance performance of the coatings is investigated.

## 2. Experimental Procedures

Al, Co, Cr, Fe and Ni metallic powders with purities higher than 99.9% were used. These powders, with the same atomic percentage, were placed in a polytetrafluoroethylene vials containing some agate balls. Mechanical milling was carried out at 300 rpm for 10 h in a planetary ball mill (PreBM-01A) with a ball to powder ratio of 10:1. The particle size of the powder ranged from 15 to 50 μm. Low carbon steel (45#) was used as the substrate material for the spraying experiment. Before deposition, the substrates were polished by SiC papers down to 150-grit. Then, the surface of the substrate was ultrasonically degreased in acetone, and then blasted by alumina powders. The size range of alumina was about 60 mesh (250 μm). Sand blasting took about two minutes for each sample. After that, the substrates were ultrasonically cleaned several times in alcohol, and dried in a drying box with a temperature of 130 °C for 2 h. The main purpose of cleaning the substrate surface many times was less oil stains and alumina particles left after sandblasting. Each substrate was cleaned five times to ensure that no stains exist on the surface. The AlCoCrFeNi coatings were deposited by an atmospheric plasma spraying (APS) system (XM-80SK, SHXM-PT, Shanghai, China) with an XM-100 spraying gun. Four different process parameters were used for the preparation of the coatings (denoted as H1, H2, H3 and H4, respectively). The power values of APS were P_H1_ < P_H2_ < P_H3_ < P_H4_. The APS parameters are shown in Table 1.

The microstructures of specimens were observed in a HITACH TM-1000 scanning electron microscopy (SEM) operated at 20 kV and equipped with an energy dispersive detector (EDAX-Phoenix). The phase structure was determined by an X-ray diffraction analysis (XRD) system (D/MAX-3BX) with Cu-Kα radiation generated at 40 kV within the 2θ range 30° and 100°. X-ray photoelectron spectroscopy (XPS) measurements were performed in a Thermo SCIENTIFIC K-ALPHA. A photoelectron emission was stimulated by a monochromatic Al K source under the condition of 150 W and initial photon energy of 1486.8 eV. The test area was 500 μm^2^ with a vacuum of 2 × 10^−9^ mbar.

The surfaces of HEAC firstly were mechanically polished to a 2000-grit SiC paper and then electrically polished. The main purpose of electrolytic polishing was to remove surface stress. The parameters were as follows—current: 20 mA maximum; voltage: 20 V; solution: 10% perchloric acid +90% carbinol; temperature: −20 °C; time: 30 s; final surface roughness of the specimens: around 0.05–0.4 μm. Nanoindentation tests were carried out in a TI 950 TriboIndenter system (Hysitron Inc., Minneapolis, MN, USA) with a Berkovich indenter. The samples were loaded to 10 mN at a constant loading rate of 2 mN s^−1^, held at the maximum load, P_max_, for 15 s, and then fully unloaded. To characterize the distribution of the mechanical property, on the cross-section of the HEAC, 18 × 9 individual nanoindentation tests were performed. The center of the matrix was the half-thickness layer of the HEAC. The error used in this paper was the limit error. First, find out the median value, in the calculation of the other data of the relative worth of size, and finally find out the maximum and minimum value. The tribological tests were performed in ball-on-disk type friction and wear tester (Germany, BRUKER, UMT TriboLab). The surface of the HEAC was polished on a 1000 mesh emery paper. A Si_3_N_4_ ceramic ball with a diameter of 5 mm was used as a rubbing pair, which was circularly rolled on to the surface of the HEAC with an applied load of 5 N for 1200 s.

The corrosion test was carried out in a 3.5 wt.% NaCl solution. An electrolytic cell with a size of 2 × 2 × 0.5 cm^3^ could fix the specimen, which ensured that the test area was 1 cm^2^. The surface was polished and cleaned with alcohol, acetone and deionized water. The polarization experiments were performed in a Potentiostat Workstation (CHI600E). All the experiments were carried out in a standard three-electrode cell system including a reference electrode of Ag/AgCl (3M KCl) solid electrode and an auxiliary electrode of a platinum sheet. The HEAC was a working electrode. During the experiment, the working electrode was corroded for 3600 s to acquire a quasistationary value of the open-circuit potential (OCP). Electrochemical impedance spectroscopy (EIS) was carried out at OCP with an amplitude perturbation voltage of 10 mV (peak to peak) and a frequency ranging from 100 kHz to 10 MHz. The potentiodynamic-polarization tests were carried out at a scan rate of 100 mV/min from an initial potential of 0.25 V vs. OCP, and stopped at the end when the current density increased to 1 mA/cm^2^. Immersion tests were performed in the 3.5 wt.% NaCl solution at room temperature for 240 h.

## 3. Results and Discussion

### 3.1. Microstructure and Phase Constitution

**Figure 1:**
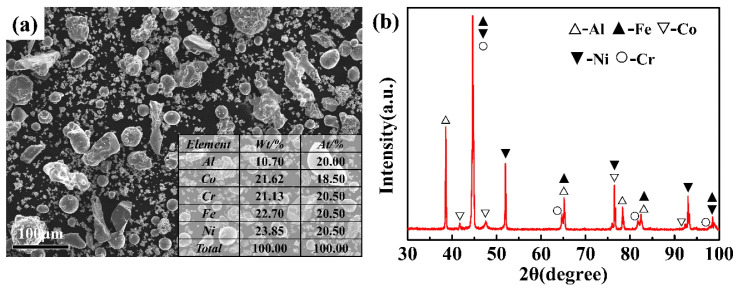
(**a**) The morphology, composition and (**b**) X-ray diffraction pattern of feedstock powder after ball milling for 10 h.

After ball milling, the morphology, composition and XRD pattern of metallic powders were measured; these are shown in Figure 1. It can be noted that the size of the powder ranged between 15 µm to 50 μm (Figure 1a). The size of the powder conformed to the size distribution of the plasma spray feedstock [30], which can directly affect the quality of the APS coating. Figure 1b shows the XRD patterns of the feedstock powders, which indicates that the pure metallic powders did not undergo alloying and oxidation during the ball milling.

**Figure 2:**
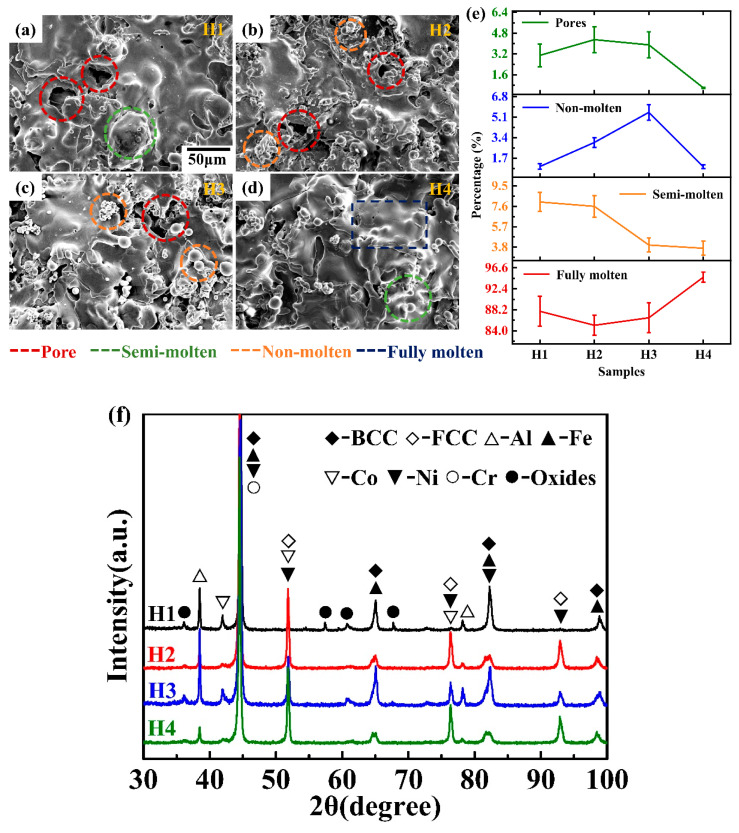
The original surfaces as a function of heat input/power: (**a**)-H1; (**b**) H2; (**c**) H3; (**d**) H4; (**e**) the volume fractions of the pores, non-molted particles, semi-molted particles and the fully-molted particles changing with the process parameters; (**f**) X-ray diffraction spectrum for the coatings.

The surface morphologies of the HEACs fabricated following the H1, H2, H3 and H4 are displayed in Figure 2a–d, respectively. Generally, during the APS process, when the feedstock powders are injected into the plasma jet, different kinetics and thermal energies are obtained by the particles due to the different particle sizes. Correspondingly, the particles experience various states, including fully-molten, semi-molten and non-molten states [24]. The semi-molten and non-molten particles can be rebounded from the coatings and exhibit some rough bulges, as shown in Figure 2a–c. The fully-molten particles form the well-flattened splats when they strike the substrate, which are shown in Figure 2d. Numerous pores can be clearly observed on the coating, except on the H4 coating. Besides, more semi-molten and non-molten particles appeared on the H1, H2 and H3 coatings, compared to that on the H4 coating. After statistical analysis of five images, the volume fractions of pores, non-molten particles, semi-molten particles and the fully-molten particles changing with the process parameters are summarized in Figure 2e. It can be seen that the volume fractions of the pores and the semi-molten particles decreased from 4.31 ± 0.90% to 0.59 ± 0.12%, and from 8.01 ± 0.89% to 3.70 ± 0.66%, respectively, when the processing parameters changed from the H1 to H4. The volume fraction of the non-molten particles increased from 1.00 ± 0.23% to 5.51 ± 0.65% and then decreased to 0.98 ± 0.15%. The quality of APS coating was determined by the temperature, flying speed and particle size of the spraying particles. The flying characteristics of the spraying particles were subject to the gas type and flow rate, arc power, nozzle structure, spraying distance, powder size distribution, etc. In the process parameters of APS, the hydrogen-gas flow rate is the most important parameter dominating the power of APS, enthalpy of the plasma arc and flying speed of the particles in the plasma arc. For the H1, H2 and H3 coatings, the hydrogen-gas flow rate gradually increased, which causes the enthalpy of the plasma arc to also increase. However, although the enthalpy of the plasma arc can also be increased, excessive gas rapidly cools the flame of the plasma, which makes the enthalpy and temperature drop. In this case, the powders were unevenly melted, and the spraying efficiency decreased, which resulted in many porosities in the H1, H2, and H3 coatings. Further increasing the hydrogen-gas flow rate induces an excessive power that causes a small amount of powder to become over-molten, which can reduce the flying speed of particles and then improve the reaction time of particles in the plasma arc, as well as the melting rate of particles. The increase in the reaction time can improve the fully-molten rate of the H1 coating. However, due to the relatively low temperature and rigidity of the plasma arc, numerous pores appear. The XRD patterns for the H1–H4 coatings are displayed in Figure 2f. The main phases of the H1 coating are BCC and FCC solutions. Some oxides and single metallic elements can also be detected. The phase structures of the H2, H3 and H4 coatings are basically consistent with those of the H1 coating. However, with the changing of the APS process parameters, the phase structures of four coatings exhibited some differences. Increasing the spraying power resulted in the intensities of the diffraction peaks from the pure Al, Fe, Co, Ni and oxides to decrease, which suggests that the percentage of pure metallic elements and oxides also decreases. It is obvious that the high spraying power enhanced the alloying process.

**Figure 3:**
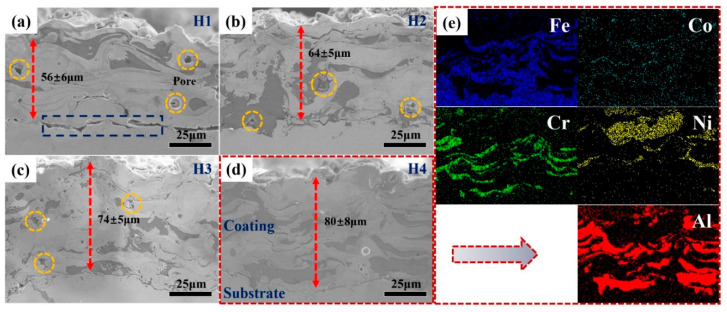
The cross-sectional morphologies of the HEACs as a function of applied power: (**a**) H1; (**b**) H2; (**c**) H3; (**d**) H4; (**e**) the corresponding elemental-distributions maps in cross section for the sample condition H4.

Figure 3a–d present the cross-sectional morphologies for the H1, H2, H3 and H4 coatings, respectively. The thicknesses are approximately 56 ± 6, 64 ± 5, 74 ± 5 and 80 ± 8 µm for the H1, H2, H3 and H4 coatings, respectively. The differences in the thickness of the four coatings indicates that the deposition efficiency of H4 is significantly higher than those of the other three coatings. As discussed above, some powders in H1, H2 and H3 were not fully melted due to the low power, which led to a low deposition efficiency. In Figure 3a, some cracks can be clearly seen at the interface between the coating and the substrate, and some pores can also be found in the H1 coating, which indicates that the coating bonded to the substrate poorly. When process parameters were changed, the cracks disappeared at the interface, but some pores still can be seen in the H2 and H3 coatings, as shown in Figure 3b,c. The pore formation can be ascribed to the loose adhesion between the spraying layers and gas escaping from the coatings. In the H4 coating, the combination of the coating and the substrate was relatively tight, and no obvious pores can be seen (Figure 3d). Figure 3a–d also exhibit the polished surfaces of four coatings, which were composed of many color contrast areas with a laminated structure. As can be seen from the above, the H4 coating had a relatively uniform structure. The elemental-distributions maps in the cross-section of the H4 coating exhibited a relatively uniform distribution (Figure 3e).

### 3.2. Nanoindentation and Frictional Wear

**Figure 4:**
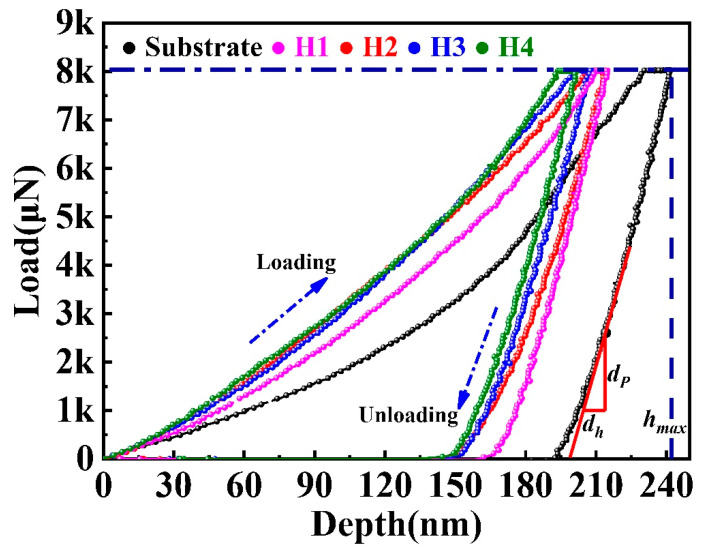
The nanoindentation loading and unloading curves for the substrate and the coatings as a function of spraying power (H1, H2, H3 and H4).

The loading-displacement curves for the substrate, and the H1, H2, H3 and H4 coatings are shown in Figure 4. The *h_max_* values of the substrate, H1, H2, H3 and H4 coatings were 242, 215, 213, 207 and 200 nm, respectively, which suggest that the coating improves the hardness of the substrate. The above results are in full agreement with the calculated hardness values of Table 2. The stiffness can be determined by calculating the initial slope of the curve (*d_P_*/*d_h_*) during unloading; then, the value of Young’s modulus can be further deduced [31]. As shown in Table 2, both the hardness and Young’s modulus of the H4 coating were significantly higher than those of the H1, H2 and H3 coatings, as well as the substrate.

**Figure 5:**
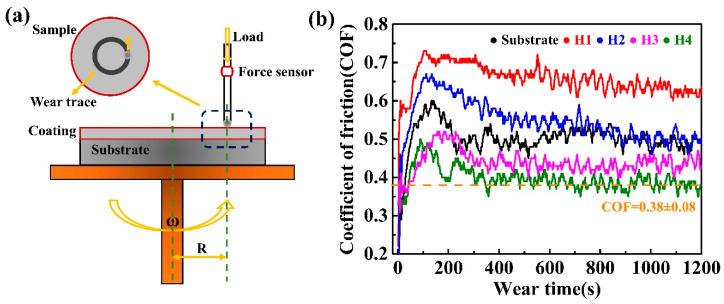
(**a**) Schematic diagram of frictional wear processing; (**b**) the coefficient of friction (COF) for the substrate and the samples with coating as a function of spraying power (H1, H2, H3 and H4).

**Figure 6:**
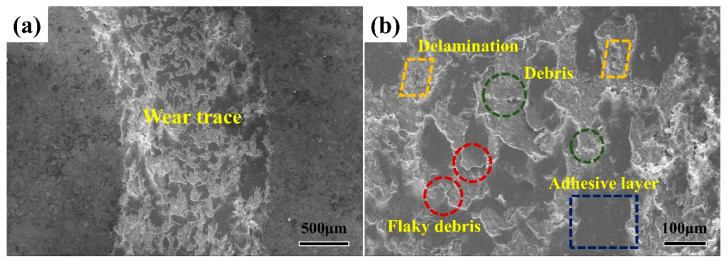

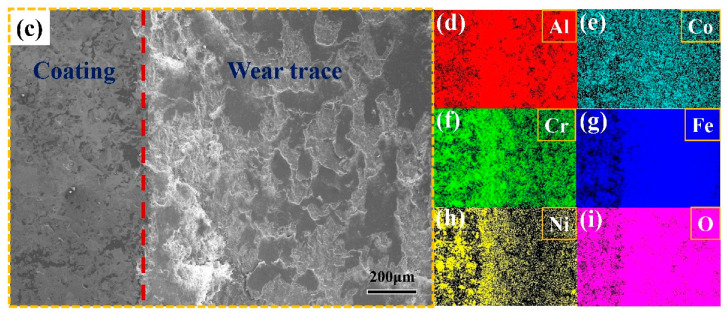
(**a**) The scanning electron images showing the wear traces for the sample with H4 coating; (**b**) higher magnification image; (**c**) the scanning electron micrograph at the junction showing both the coating and wear traces; (**d**–**i**) the elemental-distributions maps at the junction of the coating and wear traces.

A spherical disk rotation was used to investigate the frictional wear. The process diagram of frictional wear is shown in Figure 5a,b illustrates the coefficients of frictions (COFs) for the H1, H2, H3 and H4 coatings as the functions of the sliding time. The small value of the COF indicates a high wear resistance because removing the material requires massive energy [24,32,33]. With increasing the wear time, the friction coefficients increase sharply at the initial stage, i.e., from 0 to 170 s, and then gradually reach a constant value. The COFs of the substrate, H1, H2, H3 and H4 coatings were 0.49 ± 0.05, 0.63 ± 0.03, 0.50 ± 0.07, 0.43 ± 0.11 and 0.38 ± 0.08, respectively, as shown in Figure 5b. It can be clearly seen that the H4 coating exhibits the best wear resistance, i.e., the lowest COF: 0.38 ± 0.08. Thereafter, the frictional wear mechanism of the H4 coating will be further analyzed. Figure 6a shows the profile of the wear traces for the H4 coating. Four characteristic areas, i.e., the adhesive layers, debris, flaky debris and delamination were observed (Figure 6b). The formation of adhesive layers is caused by dry sliding (the rubbing-pair is a 5 mm Si_3_N_4_ ball) that leads to a high contacting temperature in worn areas. The wide adhesive worn areas and delamination are clearly observed in the wear trace of the H4 coating (Figure 6b). The composition of the adhesive layer is shown in Table 3. This demonstrates that the adhesive layer is mainly rich in Fe element that mostly comes from the substrate. Furthermore, the appearance of the flaky debris illustrates that the adhesive wear in the H4 coating was more serious than the cases in the H1, H2 and H3 coatings. In light of the theory of the adhesive wear, the adhesive material can slide between the surfaces of the friction pairs during the subsequent sliding process. Thus, with friction, the partial materials falls off the surface due to work hardening, oxidation, etc., resulting in a weight loss from the surface. The H4 coating shows severe adhesive wear, as evidenced by the intensively- plastic flow and the plowing grooves with high stress. The materials loss in the H4 coating seems to occur primarily due to its ploughing with the wedge formation because of the intensively-plastic deformation induced by the hard rubbing-pair, in particular by the oxide particles generated during the wear process itself. Figure 6c–i present the micrograph and the elemental-distributions maps for the junction areas between the coating and the wear traces. The chemical compositions of different wear-trace regions for the H4 coating are listed in Table 3. The oxygen contents in the four regions (delamination, debris, flaky debris and adhesive layer) for the H4 coating were as high as 14.27~20.22 at. %. Some oxides, for example, iron oxides, could be observed in the four areas, indicating that oxidative wear was one of the wear mechanisms.

Oxidation is challenging to manage during the APS process. The oxidization can be classified into three stages: in-flight oxidation, oxidations during the deposition and oxidations after the deposition. The oxidation of the powders mainly occurs in the in-flight process during the APS process. A high temperature and the high oxygen content of the plasma jet, regularly sweeping during the deposition of coating, are the main factors influencing the in-flight oxidation process [30,34]. The XRD pattern (Figure 2e) and the chemical compositions in Table 3 indicate that more oxides were formed in the coating and a large amount of oxides were also present at the wear traces, compared with original coating. During the wear process, the metallic oxides flaked off and randomly distributed on the surface of the coating, which effectively prohibited the occurrence of severe adhesion wear. The effect of reducing friction can adequately grease the friction process between the rubbing-pair and the coating surface because of a denudation of multi-component metal oxides and the formation of the particle-like debris on the surface of the coatings [24]. Moreover, the wear resistance is proportional to the alloy hardness, according to Archard’s law [35]; hence, H4 coating shows the best wear resistance among the investigated samples. The hardness (Table 2) and the wear resistance further confirm this hypothesis.

### 3.3. Corrosion Behavior in 3.5 wt.% NaCl Solution

**Figure 7:**
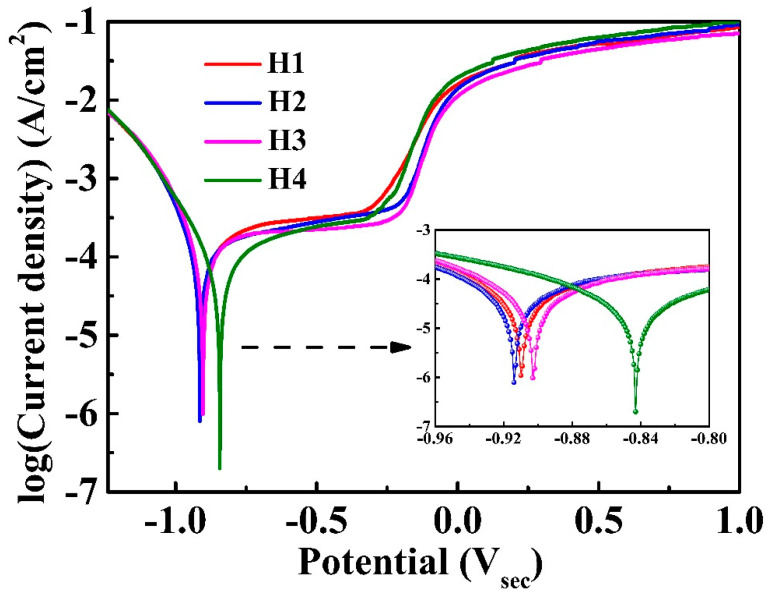
The potentiodynamic polarization curves for H1, H2, H3 and H4 coatings in the 3.5 wt.% NaCl solution.

The potentiodynamic polarization curves of the H1, H2, H3 and H4 coatings in the 3.5 wt.% NaCl solution are shown in Figure 7. Table 4 summarizes the corrosion parameters. The H4 coating demonstrates more positive corrosion potential, E_corr_, and lower corrosion-current density, I_corr_, compared to the other three coatings, suggesting an improvement in corrosion resistance. A sizeable passive region, ΔEp, indicates a good pitting resistance.

**Figure 8:**
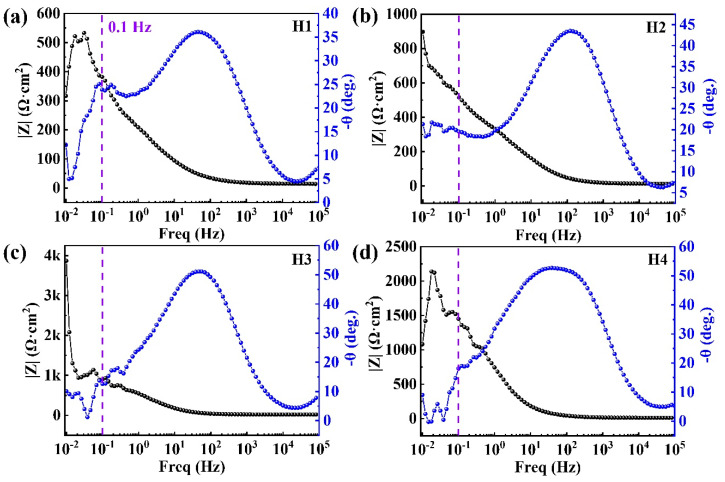
(**a**–**d**) The bode plots of H1, H2, H3 and H4 coatings in the 3.5 wt.% NaCl solution at 25 °C after 1 h of immersion.

To further analyze the corrosion mechanisms of the coatings, the passive film is characterized by the EIS and the XPS. Figure 8 exhibits the EIS plots of the passive film formed in the 3.5 wt.% NaCl solution at 25 °C after an immersion for 1 h. From the Bode plots of the HEACs, the value of |Z| at a fixed frequency of 0.1 Hz in the Bode plots is often in accordance with a polarization resistance that represents the corrosion resistance of the HEAC in the solution [36]. As shown in Figure 8, the value of |Z| at 0.1 Hz for the H4 coating is ∼1459 Ω·cm^2^; this is higher than those of the H1 (∼382 Ω·cm^2^), H2 (∼520 Ω·cm^2^) and H3 (∼887 Ω·cm^2^), which indicates that the corrosion resistance of the H4 coating is better than the other three coatings in 3.5 wt.% NaCl solution. In the Bode plots (Figure 8), with the phase angle of near zero, the value of the impedance modulus at high frequencies was almost invariable, suggesting a relatively resistive behavior. Furthermore, a maximum phase angle and a slanted value of impedance modulus at the medium and low frequencies were observed, indicating a capacitive-like behavior. In contrast to the maximum degree of the phase angle of the H1 (∼36), H2 (∼43) and H3 (∼50) coatings, the maximum phase angle of the H4 (∼53) coating was higher, indicating a significant improvement in corrosion properties.

**Figure 9:**
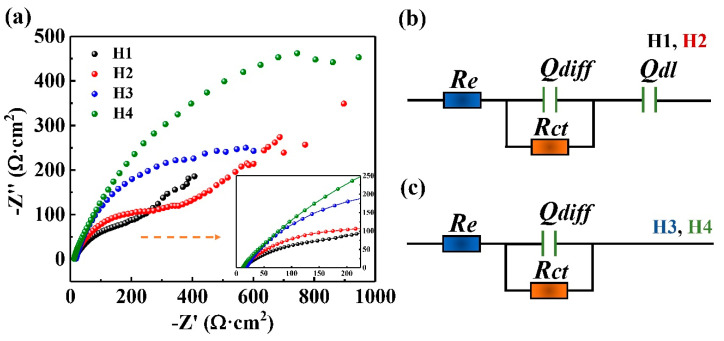
(**a**) The Nyquist plots; (**b**) the equivalent circuits of EIS for H1 and H2 coatings; (**c**) the equivalent circuits of EIS for H3 and H4 coatings in the 3.5 wt.% NaCl solution.

Figure 9a describes the Nyquist plots of the H1, H2, H3 and H4 coatings in the 3.5 wt.% NaCl solution. The diameter of the capacitive semicircle of the H4 coating was larger than those of the other three coatings, also indicating a higher corrosion resistance of the H4 as compared to those of the H1, H2 and H3 coatings. The features of H2 coating are analogous to those of H1 coating where a depressed capacitive semicircle covers most of the high-frequency regions, with a straight line appearing at the low-frequency area. The occurrence of straight line in the Nyquist plots reveals that the diffusion of oxidation products plays a negative role in the kinetics of the dissolution of surface passive film [36,37,38]. However, no apparent straight line in the Nyquist plots of the H3 and H4 coatings can be seen in the low-frequency region. The Nyquist plots for the H3 and H4 coatings under passivation conditions are characterized by a somewhat unfinished semi-circle, a feature which is potential-dependent. The diameter of the semicircle in the Nyquist plot becomes bigger with increasing the potential, suggesting a continuous growth in the corrosion resistance.

Due to the above results, the equivalent circuits (ECs) of the coatings are obviously different. The ECs used for fitting the EIS datum of the H1, H2,H3 and H4 coatings in the 3.5 wt.% NaCl solution are presented in Figure 9b,c. In the EC, *R_ct_* is the resistance of the passive film, and *R_e_* is the resistance of solution, *Q_dl_* and *Q_diff_* constant phase element (CPE) are the capacitances of film, and double-charge layer, respectively. The parameter of *Q_dl_* means the surface heterogeneity, which indicates the compactness of passive film. The CPE can explain the non-ideal capacitance response resulted from the surface inhomogeneity, roughness and adsorption effect. The impedance of CPE, *Z_CPE_*, is given [37] as

(1) $$Z_{CPE}={\left(j\omega \right)}^{-n}/Y_{0}$$

where *Y*_0_ is a proportional factor, j is the imaginary unit, ω is an angular frequency, and *n* is a CPE exponent in connection with surface inhomogeneity, locating from 0 to 1. A decreased *Y*_0_ value and an increased *n* value of *Q_f_* with decreasing roughness indicates a reduction in the porosity of the passive film. It is clear that a smooth surface can enhance the formation of a compact and protective passive film. *R_ct_*, a crucial parameter describing the corrosion resistance, greatly depends on the passive film. As shown in Figure 8d, the polarization resistance, *R_p_*, increases with decreasing the roughness, which also indicates an increase in the corrosion resistance.

Table 5 shows the EC parameters for the EISs of the H1, H2, H3 and H4 coatings in 3.5 wt.% NaCl solution at 25 °C after the immersion for 1 h. The value of *Q_dl_* for the H4 coating was larger than those of the other three coatings, suggesting that the compactness of passive film on the H4 coating is higher than those on the other three coatings. The average value of ndl is roughly 0.9, suggesting that the value of *Q_dl_* shows intermediate properties between a Warburg impedance and a regular capacitor. The *n_dl_* values of all the coatings were less than 1, suggesting that the electrochemical behavior of passive film is not a pure capacitive behavior. The charge-transfer resistances, *R_ct_*, of the H1, H2 and H3 coatings were lower than that of the H4 coatings, which suggests that the passive film formed on the H1, H2 and H3 coatings exist in more activated sites. This further causes the lower corrosion resistance of these three coatings.

**Figure 10:**
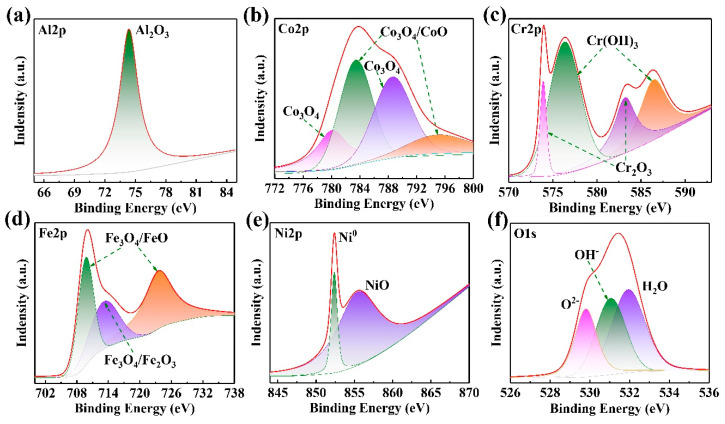
The XPS high-resolution spectra of H4 after immersion corrosion: (**a**) Al 2p; (**b**) Co 2p; (**c**) Cr 2p: (**d**) Fe 2p; (**e**) Ni 2p; (**f**) O 1s.

Regarding the best corrosion resistance of the four coatings, the H4 coating is chosen for the XPS analysis. Figure 10 shows the XPS high-resolution spectra of Al 2p, Co 2p, Cr 2p, Fe 2p, Ni 2p and O 1s for the passive film formed on the H4 coating after immersion corrosion. On the H4 coating, one Al 2p peak and four Co 2p peaks point to various oxides, i.e., Al_2_O_3_, Co_3_O_4_ and CoO (Figure 10a,b). The peaks of Cr 2p are determined to correspond Cr(OH)_3_ and Cr_2_O_3_ (Figure 10c). The Fe 2p peaks correspond to Fe_2_O_3_, Fe_3_O_4_ and FeO (Figure 10d). The peaks of Ni 2p corresponded to NiO (Figure 10e). However, the Ni-containing oxide is very susceptible to the ion bombardment during XPS profiling. Thus, the detected NiO might be induced by Ni^2+^ during argon ionic sputtering [39,40]. The O 1s spectra obtained from the passive film on the H4 coating are separated into three components (Figure 10f). The O^2−^ species consist of Co, Cr, Ni and Fe oxides. The peaks at 531.8 eV mean the OH^−^ species, evidencing a formation of metal-hydroxide [Cr(OH)_3_] in the passive film. The third peak at about 532.5 eV indicates a bound water (H_2_O) in the passive film. The bound water is considered as a capable species that can assemble the dissolving metal ions, finally forming a new film to prevent further corrosive attack. In this case, it can be inferred that the surface of the H4 coating mainly contained Al_2_O_3_, Co_3_O_4_, CoO, Cr(OH)_3_, Fe_2_O_3_, Fe_3_O_4_, FeO, NiO and the bound water (H_2_O).

**Figure 11:**
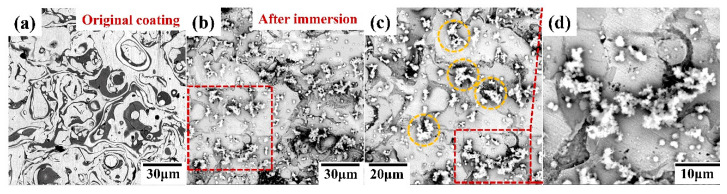
(**a**) The polished surface of H4 coating; (**b**) the H4 coating surface after immersion; (**c**) high magnification for the H4 coating surface after immersion; (**d**) oxide particles on the surface of the H4 coating after immersion in the 3.5 wt.% NaCl solution.

Figure 11 shows the surface morphologies of the H4 coating before and after an immersion in the 3.5 wt.% NaCl solution for 240 h at room temperature. Some dark and bright regions on the surface of the H4 coating can be seen in Figure 11a. Numerous layered wrapping structures were observed on the polished surface of the H4 coating. Figure 11b shows the surface morphology of the H4 coating after the immersion corrosion. As can be seen, some corrosion pits were randomly distributed on the surface. As shown in Figure 11b–d, selective corrosion occurred at the interfaces between the two phases. Many flocculent particles were generated near the pits leading to pitting corrosion. The EDS (Energy Dispersive Spectrometer) results of the flocculent particles show polymetallic oxides, which contain five metallic elements (Table 6). In addition to the flocculent particles, the corrosive surfaces also produced polymetallic oxides (Table 6). It has been demonstrated that the composition and structure of the passive films formed in the solution play a significant role in the corrosion resistance of alloys [37,41,42]. As shown above, the main compositions of the passive film are Cr and Fe oxides, which are on the H4 coating surface after the immersion corrosion. The passive film, formed at the boundary between two phases, protects poorly. Cl^−^ tends to adhere to the passive film at the boundary, which weakens and breaks the passive film, and finally results in the pitting initiation.

## 4. Conclusions

The frictional wear and corrosion resistance of the AlCoCrFeNi HEACs synthesized by atmospheric plasma spraying with four different process parameters were investigated. All the coatings were mainly composed of BCC (body-centered cubic) and FCC (face-centered cubic), containing some oxides and unsolvable metal elements. With the increasing the power of APS, the solid-solution structure was formed in the HEACs. Multi-component metal oxides were generated and randomly distributed on the surface of the coating in the process of the frictional wear process, which can prevent the serious adhesion wear from occurring. The denudation of multi-component metal oxides and the formation of the particle-like debris on the surface of the coatings can adequately grease the friction process between the rubbing-pair and the coating surface. The coating synthesized by the APS with the highest power of APS of the four coatings with different processing parameters demonstrated the most positive corrosion potential, and the lowest corrosion current density, suggesting an improvement in corrosion resistance. The improved corrosion resistance was attributed to the appearance of bound water (H_2_O) in the passive film. By changing the processing parameters, it is feasible to acquire an AlCoCrFeNi HEAC with both excellent wear resistance and outstanding corrosion resistance.

## Figures and Tables

**Table 1:** Atmospheric plasma spraying process parameters.

Samples/ Parameters	Current (A)	Voltage (V)	Ar (Slpm)	H_2_ (Slpm)	Spraying Distance (mm)	Powders Feeding Rate (g/min)
H1	550	43.6	45	0.7	117	35
H2	550	46.7	45	1.1	117	35
H3	550	51.2	45	1.9	117	35
H4	550	55.4	45	2.7	117	35

**Table 2:** Hardness and Youngs modulus values for the substrate and the four different coatings as a function of spraying power.

Parameters/Samples	Substrate	H1	H2	H3	H4
Hardness (H)/GPa	4.04 ± 0.05	4.94 ± 0.08	5.48 ± 0.05	5.83 ± 0.07	5.97 ± 0.08
Young modulus (E)/GPa	139 ± 5	118 ± 6	151 ± 5	142 ± 4	160 ± 8

**Table 3:** The chemical compositions of different wear traces regions in H4 coating (at.%).

Regions/Composition	Al	Co	Cr	Fe	Ni	O
Delamination	12.24	8.47	9.72	46.01	9.29	14.27
Debris	18.84	6.99	15.70	22.73	15.52	20.22
Flaky debris	10.41	9.20	19.34	29.77	13.29	17.99
Adhesive layer	12.79	9.11	18.21	25.44	14.67	19.78

**Table 4:** Electrochemical corrosion parameters obtained from polarization curves for H1, H2, H3 and H4 coatings tested in 3.5 wt.% NaCl solution.

Samples/Parameters	E_corr_ (V)	E_pit_ (V)	ΔE_p_	log(I_corr_) (A/cm^2^)
H1	−0.91 ± 0.04	−0.28 ± 0.04	0.63	−4.09 ± 0.18
H2	−0.91 ± 0.02	−0.23 ± 0.03	0.68	−4.15 ± 0.10
H3	−0.90 ± 0.02	−0.26 ± 0.04	0.64	−4.26 ± 0.09
H4	−0.84 ± 0.01	−0.24 ± 0.02	0.60	−4.47 ± 0.04

**Table 5:** Equivalent circuit parameter values for EIS of H1, H2, H3 and H4 coatings in 3.5 wt.% NaCl solution at 25 °C after 1 h of immersion.

Samples/Parameters	*R_e_* (Ω cm^2^)	*Q_dl_*		*R_ct_* (Ω·cm^2^)	*Q_diff_*	
		*Y*_0_ (Ω^−1^·cm^−2^s^n^)	*n_dl_*		*Y*_0_ (Ω^−1^·cm^−2^s^n^)	*n_diff_*
**H1**	**13.69**	5.11 × 10^−5^	0.90	276	5.67 × 10^−2^	0.64
H2	12.21	2.44 × 10^−5^	0.89	367	3.73 × 10^−2^	0.58
H3	17.49	1.12 × 10^−5^	0.90	649	/	/
H4	11.44	9.48 × 10^−4^	0.93	1245	/	/

**Table 6:** The chemical composition of different regions in the H4 coating after immersion in 3.5 wt.% NaCl solution.

Regions/Composition (at.%)	Al	Co	Cr	Fe	Ni	O
Immersion surface	5.73	1.85	10.39	25.59	5.46	50.98
Flocculent particles	6.49	3.33	11.92	21.07	6.32	50.87
